# Temporary effects of random positioning on the function and plasticity of proliferating monocytes

**DOI:** 10.1038/s41598-025-26941-x

**Published:** 2025-11-10

**Authors:** Shannon Marchal, Anna Dittrich, Nadine Becker, Katrin Vogel, Lisette Fickenscher, José Luis Cortés Sánchez, Stefan Kahlert, Rasika Murkar, Daniela Grimm, Marcus Krüger

**Affiliations:** 1 Department of Microgravity and Translational Regenerative Medicine, Otto-von-Guericke University, 39106 Magdeburg, Germany; 2 Department of Systems Biology, Otto-von-Guericke University, 39106 Magdeburg, Germany; 3 Department of Experimental Pediatrics, University Hospital Magdeburg, 39120 Magdeburg, Germany; 4 Institute of Anatomy, University Hospital Magdeburg, 39120 Magdeburg, Germany; 5 Core Facility Tissue Engineering, Otto-von-Guericke University, 39106 Magdeburg, Germany; 6 Research Group “Magdeburger Arbeitsgemeinschaft für Forschung unter Raumfahrt- und Schwerelosigkeitsbedingungen” (MARS), Otto-von-Guericke University, 39106 Magdeburg, Germany; 7 Department of Biomedicine, Aarhus University, 8000 Aarhus Centrum, Denmark; 8 Environmental Cell Biology Group, Department of Microgravity and Translational Regenerative Medicine, Otto-von-Guericke University Magdeburg, Universitätsplatz 2, 39106 Magdeburg, Germany

**Keywords:** Dynamic cell culture, Monocytes, Macrophages, Microgravity, Cell cycle, Mechanosensitivity, Monocytes and macrophages, Assay systems, Environmental biotechnology, Cell biology

## Abstract

The Random Positioning Machine (RPM) is used in the field of gravitational biology and space medicine. Rotational bioreactors such as the RPM create dynamic suspension cultures, providing shear stress, a crucial factor in circulatory homeostasis, but also in efficient mixing of nutrients and gases. This creates more physiologically relevant growth conditions than standard culture conditions translating the results to both microgravity and physiological systems on Earth. Immortalized monocyte-like THP-1 cells and primary blood-derived monocytes (PBMC) were cultured on the RPM for up to 7 days and evaluated for cell morphology, plasticity and functionality. For proliferating THP-1 cells, two different cell culture phases became obvious during random positioning: RPM early-phase (1–3 days) cells showed an increased responsiveness to lipopolysaccharide (LPS), plasticity for macrophage differentiation and phagocytic activity. RPM late-phase (4–7 days) cells demonstrated an impaired responsiveness to LPS, plasticity for macrophage differentiation and no phagocytic activity, emphasizing possible inhibitory effects of long-term random positioning on monocyte maturation and functionality. Interestingly, RPM cell culture had no significant effect on the phenotype of non-proliferating PBMCs, possibly related to their quiescence in G_0_ phase. These insights provide valuable information about the behavior and susceptibility of human monocytic cells under changing mechanical influences, which is important not only for gravitational research, but also for a better understanding of disease mechanisms.

**Supplementary Information:**

The online version contains supplementary material available at 10.1038/s41598-025-26941-x.

## Introduction

The development of immunocompetent in vitro tissue models is an important step in the study of physiological or disease-related processes and in the testing and evaluation of therapeutics and harmful substances^1–6^. What is already a challenge on Earth becomes even more complex in gravitational biology and space medicine. On Earth, from both the National Aeronautics and Space Administration (NASA) and European Space Agency (ESA) acknowledged, “ground-based facilities” are often used to simulate microgravity conditions for experimental approaches^7,8^. In most cases, these systems use a rotation pattern or magnetic levitation to keep a cultivated cell system in suspension. For instance, the Random Positioning Machine (RPM) works by constantly changing the orientation of the gravity vector, effectively averaging it to zero over time. The rotation of the fluid environment promotes efficient mixing of nutrients and gases within the medium, while also allowing suspension cells to experience a state analogous to free fall, thereby reducing sedimentation over prolonged periods^9^. The “simulated microgravity” conditions generated in this way are far from perfect weightlessness and have numerous side effects, which have a greater or lesser impact on the results depending on the setup and the model organism used^9–13^. Nevertheless, rotational bioreactors such as the RPM are still the means of choice for preparatory experiments and important tools in biomedical space research^10,14^. Unlike spaceflight experiments, which are logistically complex, expensive, and limited in availability, the RPM enables broader and more frequent experimentation, particularly important for preliminary studies, mechanistic insights, and hypothesis generation. Moreover, for cell types like monocytes, whose short lifespan complicates the use in real microgravity, RPM-based models provide an essential platform for studying immune responses under altered gravitational conditions. Although the shear stresses generated on the RPM (10–25 mPa) are much lower compared to the typical physiological stresses generated by blood flow^15^, which are typically between 0.1 and 9.5 Pa^16^, the RPM is gaining recognition in new research areas of mechanobiology. Mechanical loading has already been described to modulate differentiation and plasticity of human monocytes^17^. However, adequate controls are required. Therefore, the RPM is a valuable tool to characterize exactly how rotating bioreactors affect immune cell function. This will allow to understand data generated in RPM experiments and to transfer results from RPM experiments to both space microgravity and physiological systems on Earth.

Monocytes are central players in the immune system, serving crucial roles as professional antigen-presenting cells, immune sentinels, and precursors to macrophages and dendritic cells. Understanding how they respond to mechanical influences like shear stress, which is significant for circulatory homeostasis, is essential for comprehending immune processes^18^. The decisive advantage of their use in RPM experiments is that monocytes are suspension cells that are automatically kept floating by random positioning. This probably comes closest to the conditions in microgravity^9^. The human leukemia monocytic cell line THP-1 is widely used to study both monocytes and macrophages^19,20^. THP-1 cells have several technical advantages over human primary monocytes or macrophages, as they enable a very homogeneous and long-lasting stable cell culture. This makes them particularly interesting for automated long-term experiments in harsh environments (e.g. in spaceflights). THP-1 monocytes provide several further advantages over tissue-resident or peripheral blood mononuclear cells (PBMCs), being ease of access, long-term storage, faster proliferation rates, relative homogeneity with the same genetic background and the absence of “contaminating” cells^21^. However, the use of THP-1 cells also has some disadvantages. Several studies have shown that leukemic cell lines such as THP-1 cells respond differently to inflammatory stimuli than blood-derived monocytes^22,23^. Furthermore, THP-1 cells express only small amounts of CD14 and are, therefore, a poor model for blood-derived monocytes with regard to lipopolysaccharide (LPS) reactions, for example^24^. THP-1-derived macrophages can thus be considered a simplified model of primary macrophages when it comes to studying relatively simple biological processes, but they fail as an alternative source for more comprehensive immunopharmacology and drug screening programs^25^.

From a space research perspective, we focused on the feasibility and usefulness of monocyte cultures exposed to the RPM environment. Therefore, we examined and compared cultures of the model cell line THP-1 and CD14^+^ PBMCs from adult blood after random positioning for morphology, typical differentiation markers, and functionality. Dynamic culture systems for studying immune cell function are however also being used outside of gravitational biology to increase the physiological relevance of immune cell culture systems, as these systems more closely resemble the in vivo environment of suspension cells in the blood system. This way, we considered the utility of the RPM for developing better physiological cell culture systems in the study of immune cell function.

## Results

### Morphological and biological effects of random positioning on THP-1 cells

**Fig. 1:**
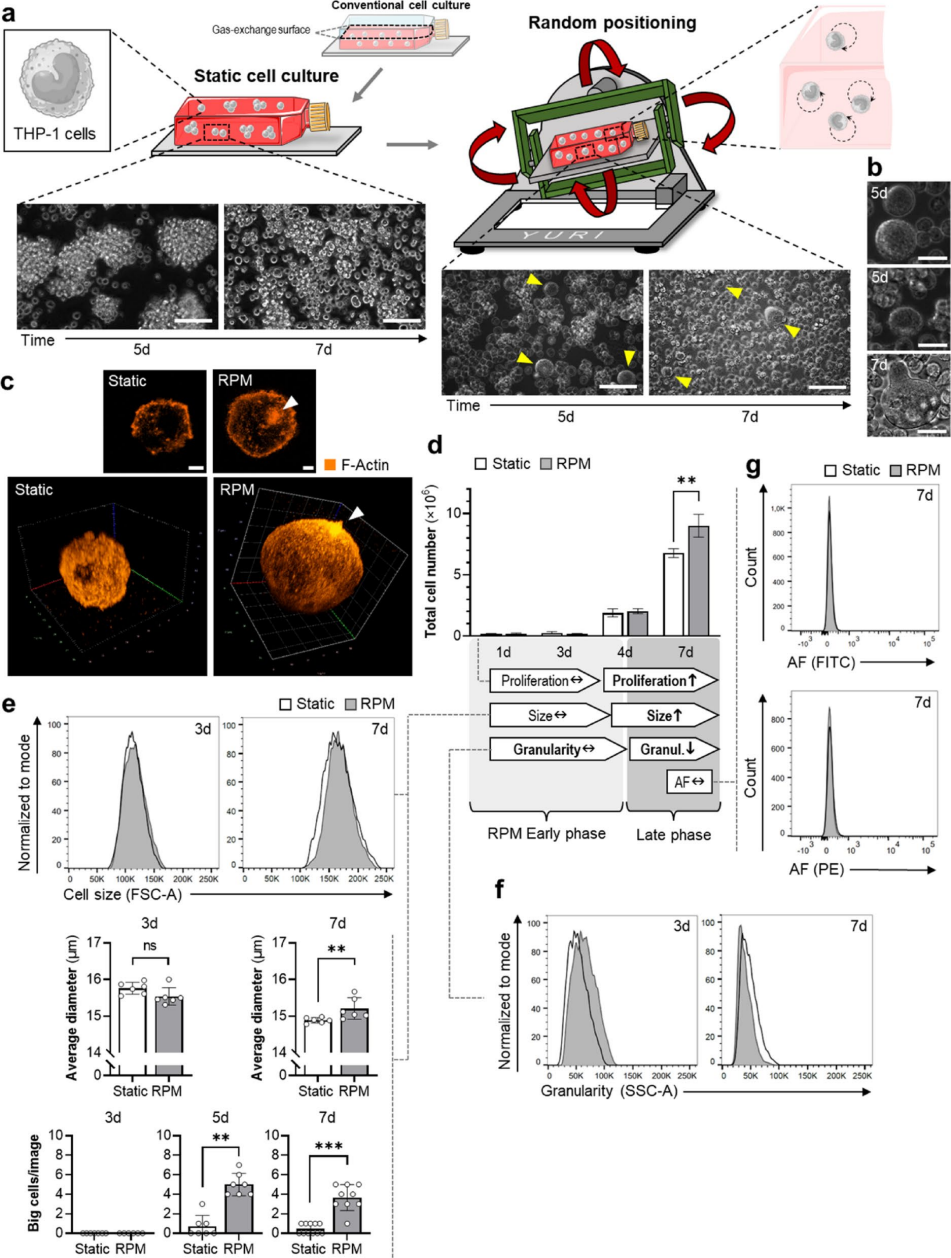
Effects of the random positioning machine (RPM) on human monocyte-like THP-1 cells. (**a**) Typical appearance of a static and a dynamic THP-1 cell culture. Random positioning of the THP-1 cells leads to an altered appearance of the cells within 5–7 d. The small section shows the presumed movement of the cells inside the culture flask due to RPM rotation. Scale bars: 100 μm. (**b**) Close-up images of individual larger cells with altered morphology on the RPM. Scale bars: 100 μm. (**c**) Immunofluorescence of F-actin after 3 d shown as cross-section (small pictures, scale bars: 5 μm) and 3D model of a hyper stack (big pictures). (**d**) Total cell numbers during a 7 d experiment (*n* = 5) (below) Observed cell culture effects on the RPM. (**e**) Effects on cell size analyzed by flow cytometry (top, *n* = 5) and cell viability analysis (middle, *n* = 6). Number of large cells per microscopic image (20×). (**f**) Effects on cell granularity analyzed by flow cytometry (*n* = 5). (**g**) Effects on autofluorescence (AF) in the FITC and the PE channels. Non-parametric Mann–Whitney U test ** *p* ≤ 0.01, *** *p* ≤ 0.001, ^ns^ non-significant. FSC-A: forward scatter area; SSC-A: sideward scatter area. Parts of the figure were drawn by using pictures from Biorender.com and Servier Medical Art.

To assess the effects of random positioning on human immune cells, we first cultured monocyte-like THP-1 cells on the RPM for up to 7 days. Note, that cell culture flasks were completely filled with medium for both static and RPM conditions, while conventional cell culture is done in a partly filled culture flask with medium/air contact (Fig. 1a). Compared to a static cell culture, the RPM-exposed cells showed a changed morphology after 5 to 7 days: qualitatively they seem to grow less in aggregates, and individual larger cells with a progressively smoother shape were visible (Fig. 1a, arrows; Fig. 1b). F-actin staining in large THP-1 cells from RPM cultures revealed the typical marginal localization, as also observed in statically cultured cells. In addition, these cells displayed actin accumulation within outward protrusions (Fig. 1c, arrow). In our estimation, such protrusions were predominantly observed in RPM cultures, infrequently under static conditions, but not in conventional cell cultures (Supplementary Fig. S1).

In addition, the rotated cell culture contained a larger number of cells after 7 days (+ 30%), indicating a higher cell proliferation on the RPM, which starts only after 4 days of rotation (+ 10%) (Fig. 1d). Although observations after 5 to 7 days showed individual larger cells on the RPM (Fig. 1b and e, lower panel), flow cytometry results of the entire cell population did not indicate a significant change in cell size between the two culture conditions. However, a small population of cells with larger forward scatter was observed in the RPM group (Fig. 1e, upper panel), that confirms presence of individual larger cells. Furthermore, independent automated cell counting analysis confirmed our visual observations and showed a slightly increased average diameter of the THP-1 cells on the RPM after 7 days, but not after 3 days (Fig. 1e, middle panel). Flow cytometry findings showed a short-term increase in granularity of THP-1 cells (1–3 days) on the RPM compared to static conditions, followed by a decrease after 7 days (Fig. 1f). It is often reported that monocyte differentiation is accompanied by an increased autofluorescence of the cells^21,26^. In our experiments, the autofluorescence intensities in the FITC and PE channels of THP-1 cells showed no significant change between the two groups (Fig. 1g) and were therefore complemented by an additional analysis of differentiation markers at a later time point to rule out a false-negative result as conclusions about differentiation should not be based on autofluorescence alone.

### Effects of random positioning on the differentiation capability of THP-1 cells

Various biophysical properties of a cell (such as cell size, cell shape and distribution of intracellular structures) represent the emergent properties of cell phenotype and function^27^. Since changes in these properties can indicate monocyte differentiation^21^, we next looked at the differentiation capability of THP-1 cells. In preliminary experiments, we chemically differentiated THP-1 cells into M_0_ macrophages using PMA (phorbol 12-myristate 13-acetate), which led to an optical change in cell morphology under the microscope (Supplementary Fig. S2a). Differentiation success was further determined by the cellular expression of activation antigens (CD14, CD71) as evidence of the maturation of THP-1 cells^28,29^. Both activation markers CD14 and CD71 increased in PMA-treated THP-1 cells (Supplementary Fig. S2b).

**Fig. 2:**
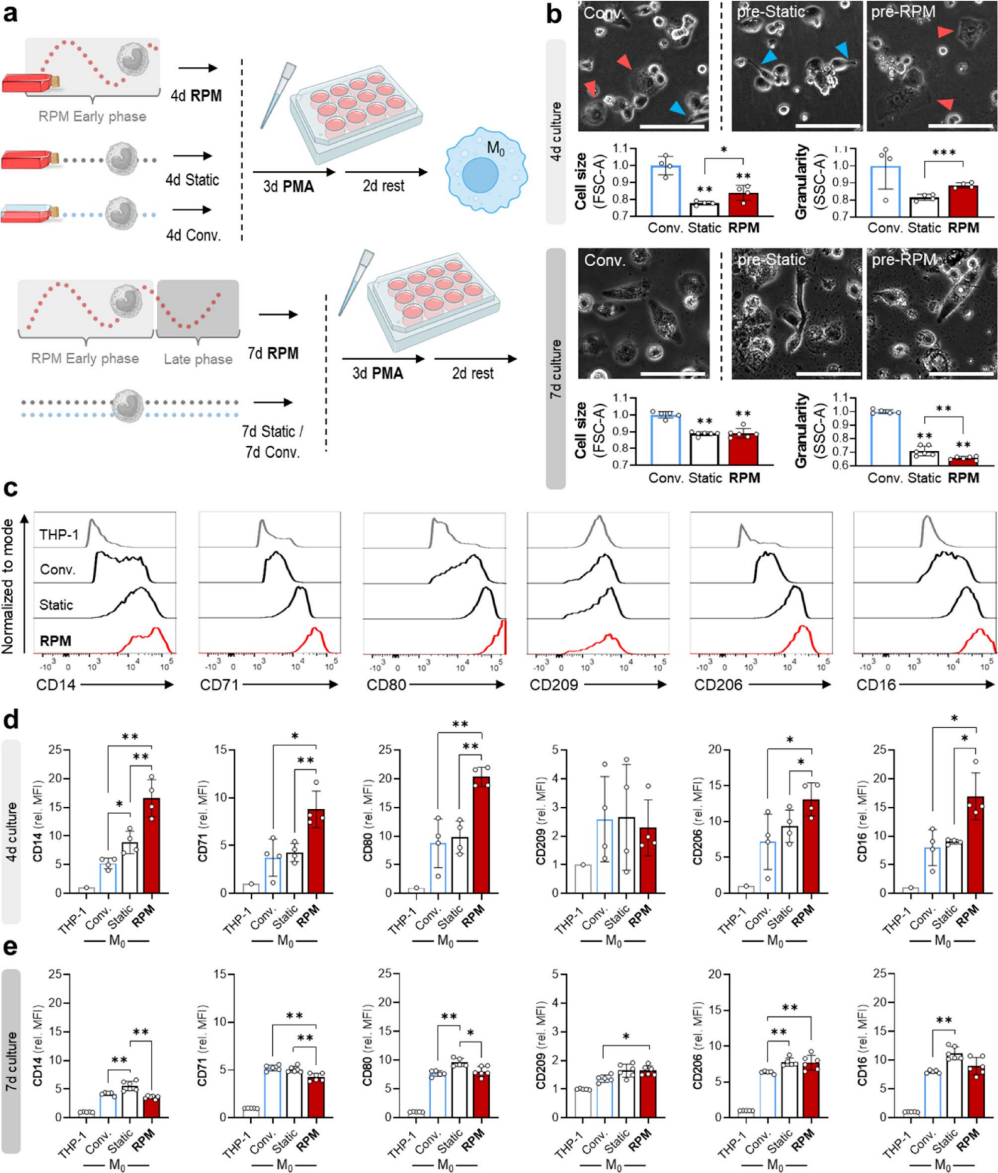
Effects of cell culture conditions on the response of THP-1 cells to PMA stimulation. (**a**) Schematic overview of experimental timeline. (**b**) Effects of pre-culture conditions on cell size and granularity of M_0_ macrophages (top *n* = 4, bottom *n* = 6). Scale bars: 50 μm (20×). (**c**) Marker expression on PMA-differentiated M_0_ macrophages after 4 d under different culture conditions (*n* = 4). (**d**) Mean Fluorescence Intensity (MFI) of CD14, CD16, CD71, CD80, CD206 and CD209 as determined by flow cytometry after 4 d (*n* = 5). (**e**) and after 7 d (*n* = 6) under different culture conditions. Independent sample t-test (*n* = 4) or non-parametric Mann–Whitney U test (*n* ≥ 5) * *p* ≤ 0.05, ** *p* ≤ 0.01, *** *p* ≤ 0.001. Parts of the figure were drawn using pictures from Biorender.com and Servier Medical Art.

Next, we pre-cultured the THP-1 cells on the RPM for 4 and 7 days prior to PMA-differentiation. Control cells were cultured under conventional (conv.; medium/air contact) or static (static; medium filled flask) conditions for the same amount of time before PMA differentiation (Fig. 2a). Differentiation success was confirmed by optical inspection. Differentiated cells attached to the bottom of the well plate and showed either an elongated or ‘egg-shaped’ morphology. Interestingly, static cultured cells showed a more elongated morphology (Fig. 2b, blue arrows) while cells on the RPM became more ‘egg-shaped’ (Fig. 2b, red arrows). PMA-treated THP-1 cells, after 4 days on the RPM, increased more in size and granularity compared with static cell culture (Fig. 2b, top). However, this difference was not observed anymore after 7 days (Fig. 2b, bottom). Changes in granularity could thus be a remaining culture effect independent of PMA treatment, as this was already observed in cells not treated with PMA (Fig. 1f). Interestingly, conventional cell culture with medium/air contact resulted in better differentiated M_0_ macrophages as seen by the largest increase in cell size and granularity with a mixed morphology in the response to PMA (Fig. 2b).

Macrophages and their distinct functional phenotypes depend on environmental stimuli and are characterized by plasticity^30^. Thus, we additionally analyzed the expression of various surface markers for macrophage differentiation. The selected marker panel consisted of activation markers CD14, CD71 and phenotypical antigens such as CD80, CD209, CD206 and CD16^31–33^. Under all analyzed culture conditions, 4-day PMA-differentiated cells revealed an increased surface expression of both activation markers and CD16, CD80 and CD206, but not CD209 (Fig. 2c). Interestingly, cells pre-cultured on the RPM for 4 days showed the highest expression of these antigens, while cells cultured under conventional and static conditions express comparable amounts of analyzed surface markers (Fig. 2d). This suggests an increased plasticity in response to PMA of cells cultured on the RPM. After 7 days, these effects could not be observed anymore. Instead, RPM-cultured cells showed a slightly reduced activation status as marked by reduced CD14 and CD71 expression. Phenotypical antigens were either unchanged (CD80, CD16) or slightly increased (CD206, CD209) compared to the conventional culture group. Moreover, static cultured cells showed the highest expression levels for CD14, CD16 and CD80 (Fig. 2e). These findings suggest a strong influence of the type and duration of culture systems used on monocyte differentiation and macrophage plasticity. While conventional cell culture with medium/air contact seemed to be superior to the completely filled flask conditions in terms of morphology, the RPM induced an increased expression of important phenotypical and functional surface markers, after four days of culture. This suggests that the cells potentially benefit from transiently rotated culture conditions, as evidenced by an increased responsiveness and activation to PMA.

### Effects of random positioning on the phenotype of THP-1 cells

**Fig. 3:**
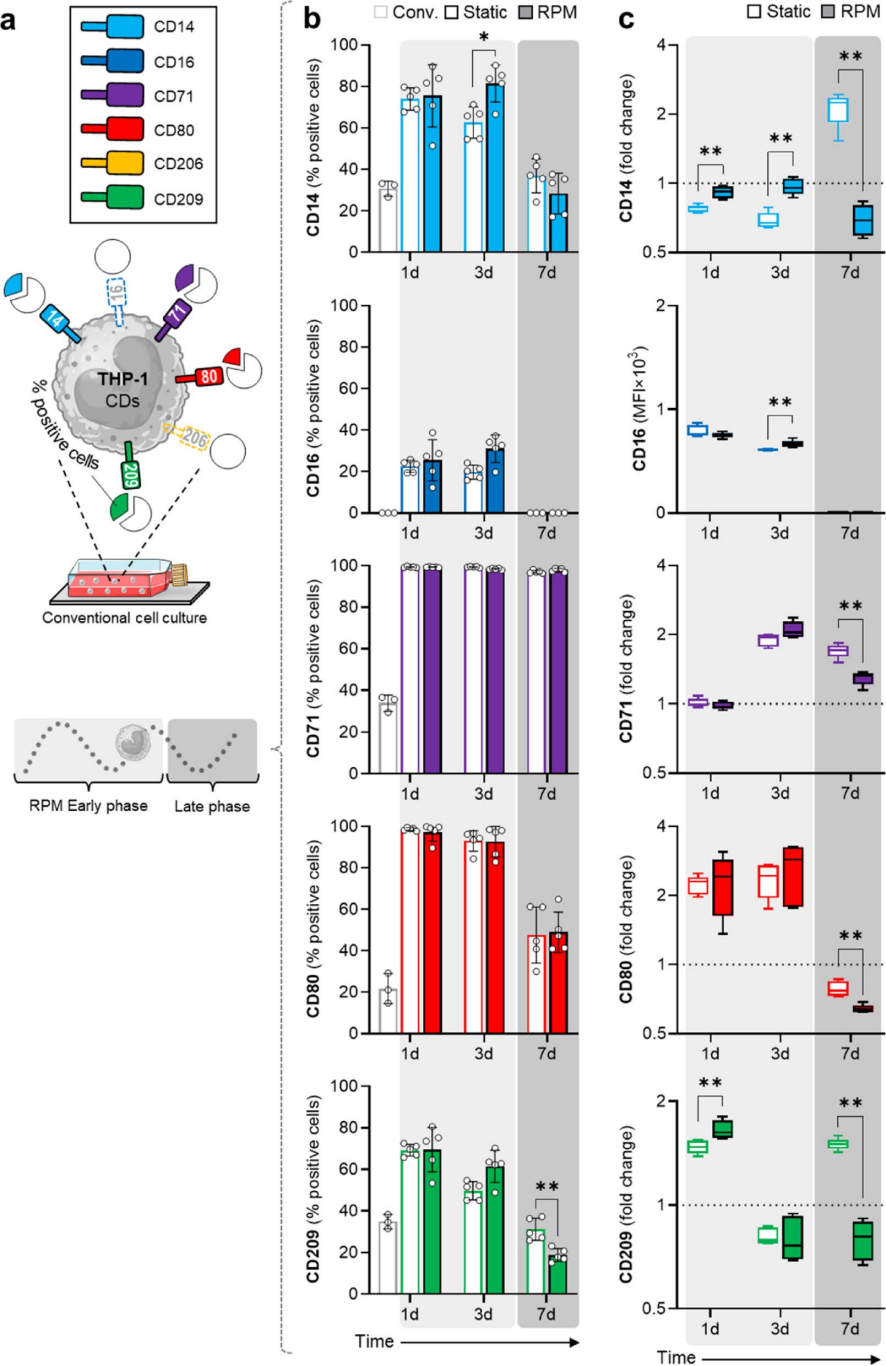
Effects of the RPM on cluster-of-differentiation (CD) markers expressed by THP-1 cells. (**a**) Marker panel and basal expression on THP-1 cells. The percentage of CD^+^ THP-1 cells in normal cell culture is shown in pie charts. Pie charts correspond to 3b grey bar plots (conv.). (**b**) Percentage of cells expressing certain surface markers under different culture conditions. (**c**) Mean Fluorescence Intensity (MFI) of CD14, CD16, CD71, CD80 and CD209 as determined by flow cytometry (*n* = 5); conventional cell culture (dotted line) corresponds to 1. Non-parametric Mann–Whitney U test * *p* ≤ 0.05, ** *p* ≤ 0.01. Parts of the figure were drawn using pictures from Biorender.com and Servier Medical Art.

The characterization of the cell phenotype could also provide further insights into the previously observed PMA-independent changes by RPM. Thus, the panel consisting of the surface markers CD14, CD16, CD71, CD80, CD206 and CD209 was used to analyze activation of THP-1 cells independent of PMA. Only 30% of the THP-1 cells expressed the monocyte-typical marker CD14 under conventional cell culture. Additionally, THP-1 cells cultured under conventional conditions did not express CD16 and CD206, and only partially expressed CD71 (34%), CD80 (22%) and CD209 (35%) (Fig. 3a).

Next, the functional phenotype was compared between static and dynamic culture conditions after 1, 3 and 7 days. The percentage of CD14^+^ and CD16^+^ THP-1 cells strongly increased after 3 days of rotated or static cell culture (~ 80% and 60%, respectively) and decreased again after 7 days (~ 30%; Fig. 3b). This observation was partly reflected in the expression levels of CD14 on individual cells, which was higher in RPM-cultured cells than in static cultured cells after 1 and 3 days but lower after 7 days. RPM-exposure also increased the surface expression levels of CD209 and CD16 compared to static culture after 1 and 3 days, respectively (Fig. 3c). The transient increase in CD14 and CD16 expression after 3 days on the RPM indicates a transient more physiological state than under conventional or static conditions. It is important to note that the largest differences in marker positive cells (Fig. 3b) were observed between completely medium filled culture conditions (static/RPM) and conventional cell culture, especially after 3 days. For instance, the frequency of CD71^+^ cells increased to approximately 95–100% in both conditions when cells were cultured in completely medium filled flasks. Similar trends were observed for the other markers, which increased in cells cultured in completely medium filled flask conditions after 3 days, which leads us to believe that other factors besides rotation, such as the physico-chemical environment, have a large influence on THP-1 phenotype that should not be ignored.

After 7 days, the percentage of CD14^+^ and CD209^+^ cells was comparable between all culture conditions. Notably, the percentage of CD209^+^ cells decreased more in RPM-cultured THP-1 monocytes than in static cultured cells after 7 days. The percentage of CD71^+^ and CD80^+^ cells remained elevated upon cells cultured with both completely medium filled flask conditions (Fig. 3b).

Interestingly, static-cultured THP-1 monocytes after 7 days expressed higher levels of surface antigens CD14, CD71, CD80 and CD209 compared to RPM-cultured THP-1 cells, and higher levels of surface antigens CD14, C71 and CD209 compared to conventional cell culture (Fig. 3c). This again highlights that length and type of THP-1 culture system is critical for optimal outcomes of differentiation.

### Effects of random positioning on the viability of THP-1 cells

**Fig. 4:**
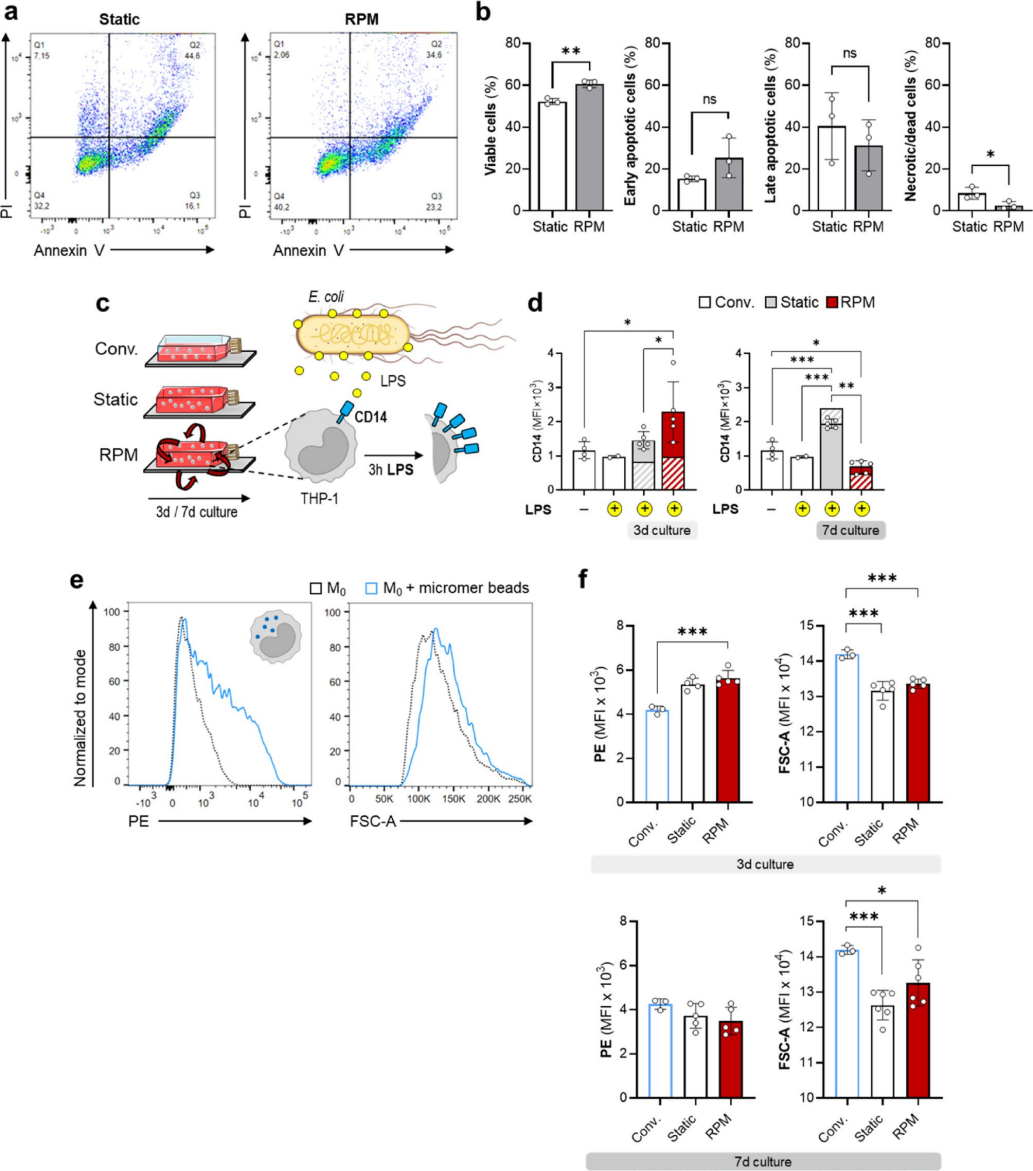
Effects of an RPM cell culture on the viability and functionality of THP-1 cells. (**a**) Annexin V and PI staining in THP-1 cells cultured for 7 d under static or RPM conditions. The fourth quadrant represents living cells (annexin V, PI negative), the third quadrant early apoptotic cells (annexin V positive, PI negative), the second quadrant late apoptotic (annexin and PI positive) and the first quadrant necrotic or dead cells (annexin V negative and PI positive). (**b**) Percentages of viable, apoptotic and necrotic cells of different individual experiments (*n* = 3). (**c**) Effects of lipopolysaccharide (LPS) stimulation on THP-1 cells. Despite low basal expression of CD14, CD14^+^ THP-1 cells should upregulate CD14 in response to LPS stimulation. (**d**) Mean Fluorescence Intensity (MFI) of CD14 in response to LPS after different culture conditions (*n* = 2–5). The hatched bars show CD14 expression without LPS, the filled bars show the effect of LPS on CD14 expression. (**e**) Micromere particle uptake by THP-1 derived M_0_ macrophages. Phagocytosis was confirmed by an increase in cell size and autofluorescence in the PE-A channel. (**f**) Phagocytosis after different culture conditions (3–7 days) reflected by changes in autofluorescence (PE) and cell size (FSC-A). Independent sample t-test (*n* < 5) or non-parametric Mann–Whitney U test *(n* ≥ 5*)*, * *p* ≤ 0.05, ** *p* ≤ 0.01, *** *p* ≤ 0.001, ^ns^ non-significant. Parts of the figure were drawn using pictures from Biorender.com and Servier Medical Art.

The extent to which the rotated cell culture influences the viability of THP-1 cells after 7 days was tested with an annexin V/propidium iodide (PI) staining (Fig. 4a). A significant difference between static and dynamic cell culture was observed with a higher frequency of viable cells and a lower frequency of necrotic/dead cells on the RPM (Fig. 4b).

### Effects of random positioning on LPS responsiveness of THP-1 cells

Lipopolysaccharide (LPS) is a potent stimulant of the innate immune system^34^. Monocyte responsiveness to LPS, as measured by upregulation of CD14, is a common functional test of monocytes (Fig. 4c). However, because of the low basal expression levels of the LPS receptor CD14 on THP-1 cells in conventional culture (Fig. 3b), these cells are far less responsive to LPS than PBMCs^19^. Since cell culture conditions influence the CD14 surface expression of THP-1 cells (Fig. 3b), it was next analyzed whether THP-1 cells cultured under static and RPM conditions are responsive to LPS and thus to test if the observed phenotypical changes are significant on a functional level.

THP-1 cells, after 3 days on the RPM, responded to LPS with an increased expression of CD14 (red bar, Fig. 4d), whereas cells from conventional (white bar) and static cell cultures (grey bar) did not and were, therefore, comparable to untreated THP-1 cells (Fig. 4d). After 7 days, no responsiveness to LPS could be observed. While it seems that the static cell culture expresses significantly more CD14, the upregulation of CD14 was also observed in cells not treated with LPS (hatched bar) and is thus rather a consequence of culture conditions.

### Effects of random positioning on phagocytic activity of THP-1 cells

Phagocytosis is one of the first lines of defense against invading microorganisms and an intrinsic property of monocyte-macrophage function^35^. Synthetic polymeric particles have been widely used to study phagocytosis^36–38^. A preliminary experiment confirmed micromere particle uptake by PMA-differentiated conventionally cultured THP-1-derived macrophages (M_0_), observed by an increase in fluorescence in the PE channel and cell size (Fig. 4e).

Next, we tested whether the different culture conditions influence the phagocytic activity of M_0_ macrophages. No significant differences could be observed between static and RPM pre-cultured cells. However, static and RPM pre-cultured cells showed an increased phagocytic activity indicated by higher fluorescence after 3 days compared to conventional cell culture (Fig. 4f, top). This effect was not observed anymore after 7 days (Fig. 4f, bottom). Interestingly, while conventional cell culture showed a concomitant increase in cell size with an increase in fluorescence (Fig. 4e), cells cultured under RPM or static conditions were significantly smaller than conventional cultured cells exposed to synthetic polymeric particles (Fig. 4f).

### Effects of random positioning on CD14^+^ PBMCs isolated from blood

**Fig. 5:**
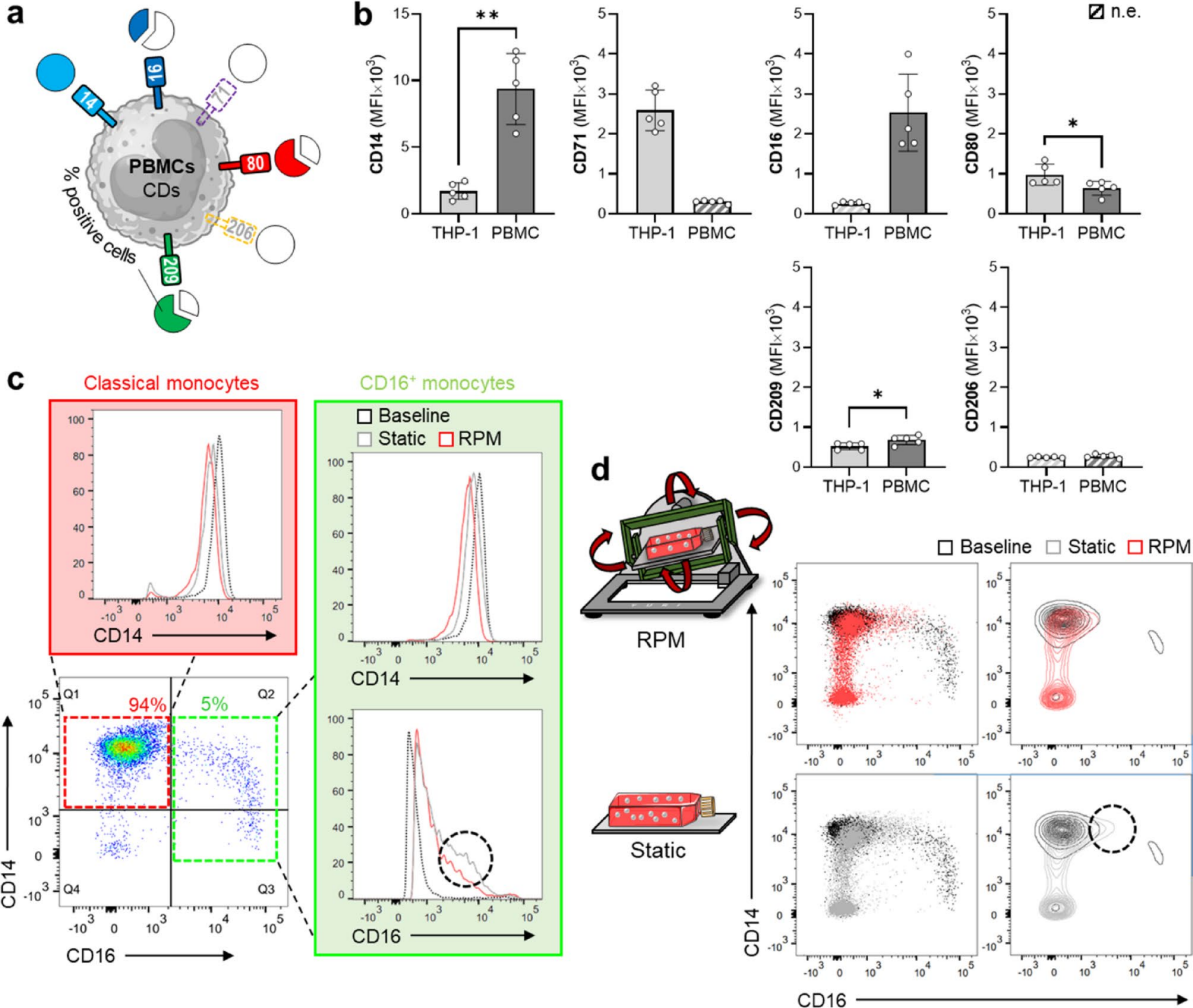
Comparison of dynamic culture effects on THP-1 monocytes and CD14^+^ PBMCs from blood cones. (**a**) Baseline percentage of CD^+^ PBMCs is shown in pie charts (*n* = 5). (**b**) Comparison of surface markers between THP-1 monocytes and blood-derived monocytes at baseline (conventional cell culture or freshly isolated). (**c**) Monocyte subsets based on relative CD14 and CD16 expression. Flow cytometry dot plot showing the classical and CD16^+^ (intermediate and non-classical) subsets of blood-derived monocytes at baseline. Representative flow cytometry histograms showing marker expression of CD14 and CD16 in PBMC under different culture conditions after one day (*n* = 5, independent donors), divided for each subset. The red square represents the classical monocytes. The green square represents the CD16^+^ monocytes. The black dotted circle highlights a small population of cells within the static culture that have a relatively high CD16 expression. (**d**) Representative flow cytometry dot plots showing marker expression of CD14 and CD16 in PBMCs under different culture conditions after one day compared to baseline expression levels (*n* = 5 independent donors). Black dotted circle highlights a small population of cells with a relatively large CD16 expression. Non-parametric Mann–Whitney U test, * *p* ≤ 0.05, ** *p* ≤ 0.01. n.e., not expressed; PBMC, peripheral blood mononuclear cell. Parts of the figure were drawn using pictures from Biorender.com and Servier Medical Art.

Freshly isolated CD14^+^ PBMCs (within 3 h of isolation) expressed basal levels of CD14 (100%), CD16 (38%), CD80 (66%) and CD209 (69%) antigens on their surface (baseline measurement; Fig. 5a). First, a comparison was made of basal marker expression between THP-1 monocytes and blood-derived monocytes. Blood-derived monocytes express higher levels of CD14, CD16 and CD209 antigens, while THP-1 monocytes express higher levels of CD71 and CD80 antigens (Fig. 5b). There was no difference in CD206.

Next, we gated for monocyte subsets by their level of CD14 and CD16 expression. There are three main monocyte subsets recognized: classical (~ 85%) that highly express CD14 but not CD16 (CD14^++^CD16^−^), intermediate (~ 5%) that highly express CD14 and concomitant expression of CD16 (CD14^++^CD16^+^), and non-classical (~ 10%) that express low levels of CD14 but high levels of CD16 (CD14^+^CD16^++^). However, the notion of intermediate monocytes is of current debate^39^. Thus here, no distinction between intermediate and non-classical monocytes was made, rather referring to them collectively as CD16^+^ monocytes. The freshly isolated PBMCs showed a subset distribution of ~ 94% classical monocytes and ~ 5% CD16^+^ monocytes (Fig. 5c).

Next, the PBMCs were cultured under completely medium filled flask conditions (static/RPM) for 1 and 3 days and analyzed for surface marker expression. The RPM culture had no impact on blood-derived monocyte surface marker phenotype compared to completely medium filled static flasks (data not shown). These findings were surprising since we hypothesized that especially primary immune cells would benefit more from culture conditions that more closely resemble the physiological environment. However, it is worth mentioning the small non-significant changes observed for CD14 and CD16 expression. While CD14 expression was reduced under static and RPM conditions, expression of CD16 was increased by these conditions compared to baseline (Fig. 5c). It is well documented that the CD14^+^ PBMC subset progression inside the blood system follows a subtle decrease in CD14 expression with concomitant increase in CD16 expression over time^32,40^. In both classical and CD16^+^ monocyte subsets the decrease in CD14 expression was more pronounced in the RPM group than under static conditions. However, more cells with a high CD16 surface expression were observed in static conditions compared to the RPM conditions (Fig. 5c). Another interesting observation to note was a population shift towards CD14^–^ and CD16^–^ cells that appeared in both completely medium filled flask conditions (Fig. 5d). This suggests that the PBMCs did not respond well to the culture conditions inherent of RPM research (i.e., complete filling of the culture flasks with medium). Due to the low cell number after 3 days of culture, hardly any meaningful analyses could be performed. However, based on the limited data available, it can also be assumed that the RPM has no effect on a three-day PBMC culture compared to static cell culture (data not shown).

## Discussion

While most RPM experiments were so far performed with monolayers of adherent cells, we used suspension cells to study the effects of random positioning. We were able to show that monocyte culture on the RPM is possible, and that rotation has no detrimental influence on the cells.

In this study, RPM-induced effects differed between the immortalized THP-1 cell line and the primary monocytes. Although this suggests that immortalization status may influence cellular responses, the comparison was limited to one cell line and one primary source. These findings should therefore be viewed as preliminary, underscoring the need for broader studies to determine whether this represents a generalizable phenomenon. Nonetheless, these findings provide new insight into the dynamic culture behavior of circulating blood cells and highlight the potential of gravitational biology research in human systems.

### RPM culture of THP-1 monocytes

The THP-1 phenotype changed in response to RPM culture by an increase in CD14^+^ cells, a typical marker for monocyte-macrophage differentiation. CD14 expression reflects the culture conditions and serves as a marker for response to PMA and LPS^41^. We observed a larger LPS- and PMA-induced increase in CD14 expression in monocytes with more basal CD14 antigen levels after 3 days (Figs. 2 and 4). However, we cannot dismiss the large differences between completely medium filled flask conditions and conventional cell culture conditions (i.e., medium/air contact) in terms of THP-1 phenotype.

It is worth to emphasize the physico-chemical properties inherent to RPM research (i.e., changes in oxygenation and pH) as they perhaps determine the observed results more than the mechanical environment. Previous findings have demonstrated that culture conditions significantly influence THP-1 phenotype and response to PMA stimulation^41^. Variations in culture conditions include media composition, cell density, concentration gradients and duration. For instance, the RPM’s rotation mixes the culture medium, leading to a more uniform distribution of nutrients and gases, unlike static conditions, where cells settle and experience uneven local concentrations. This difference may explain the increased proliferation observed under RPM conditions (Fig. 1d) as static cultures are more susceptible to local nutrient depletion. While we did not directly assess medium oxygenation or pH in our experiments, previous work with adherent thyroid cancer cell lines has demonstrated 5–10% differences in oxygen levels between RPM-completely filled flasks and static-completely filled flasks^42^. We expect similar to lower differences in our experimental set-up due to the lower cell number that was used. Nonetheless, future RPM-based studies should systematically document oxygenation and pH parameters for each individual cell line, as these factors may contribute to variability in cellular responses. Lastly, previous studies have reported a stronger response to PMA in high-density cultures compared with low-density cultures, the latter of which did not exhibit an increase in surface CD14 expression. In the present study, although a low cell density was used—that was comparable to the previously described low-density condition—the response observed more closely resembled that of high-density cultures. These findings indicate that cell density may have played only a minimal role, if any, in the results obtained^41^. However, these observations may help explaining the discrepancies reported in the literature and emphasize the need for standardized protocols and detailed methodological reporting to ensure reproducibility and comparability across studies using RPM widely adopted model system^21,43–48^.

Characterization of cell surface proteins expressed is informative not only to improve understanding of phenotype but may also provide biological insights into function. Aldo et al.^41^ showed that high-density monocyte cultures with basal CD14 expression are more sensitive to PMA as observed by an increase in CD14^+^ cells and pro-inflammatory cytokine production as opposed to a low-density culture with no basal CD14 surface antigens. Here we observed a more egg-shaped’ morphology after 3 days of RPM-precultured, PMA-differentiated macrophages (Fig. 2), indicating a potential ‘pro-inflammatory’ phenotype on the RPM. It is important to note, that surface proteins only account for a small fraction of the total proteins in a cell, and cell attributes may not correlate with the presence or expression level of surface antigens^49^. Kim et al.^50^ showed lower CD14 expression in PMA-differentiated macrophages in response to LPS compared to untreated macrophages. The opposite was observed in THP-1 cells with lower basal CD14 surface antigens compared to monocyte-derived-macrophages, indicating that the differential susceptibility might not be explained by LPS-related receptor, CD14 alone.

Morphological changes observed between dynamic and static cell cultures of THP-1 monocytes and subsequent differentiation are likewise related to the functional activity of the cells. J-111 monocytes exposed for 1 h to modelled RPM conditions compared with static controls showed a reduced filamentous density of the F-actin network with actin stress fibers accumulated in continuous sub-plasmatic bundles^51^. Similar results were obtained from real microgravity research on board the ISS^52^. In addition, Thiel et al.^53^ demonstrated rapid changes in macrophage morphology and cytoskeletal organization under microgravity conditions on a suborbital rocket flight. In the present work, we found that RPM-cultured THP-1 monocytes had a rounder cell shape compared to static cultured controls (Fig. 1). Static-cultured THP-1 cells resembled a more activated state, as described by an irregular shape with projections called pseudopodia^54^. All these findings indicate that the microgravity environment can lead to significant changes in cellular morphology, as observed through cytoskeleton rearrangement in monocytes. This could potentially impair monocyte adhesion and migration abilities, which are important for monocyte function and immune responses. In addition, changes in the cytoskeleton may significantly affect phagocytic activity, including the formation of pseudopodia and bead engulfment with the formation of a phagosome in the cytoplasm. Macrophage phagocytosis can be induced by various types of receptors, their involvement determined by the microenvironment and potential for macrophage differentiation and activation^55^. Our findings highlight an interaction between macrophage plasticity, differentiation success, and phagocytic activity of THP-1-derived macrophages after short-term dynamic cell culture.

Differing early and late effects of RPM cell culture were observed in the leukemic THP-1 monocytes, a popular model cell line. THP-1 monocytes seem to benefit from random positioning as seen by an increased responsiveness to LPS, plasticity for macrophage differentiation and phagocytic activity, only after 3 days. These results follow the physiological perspective that monocytes might benefit from a culture system that more closely resembles the physiological environment. Late timepoints impaired responsiveness to LPS, plasticity for macrophage differentiation and no phagocytic activity, emphasizing possible inhibitory effects of long-term random positioning on monocyte maturation and functionality. This observation either reflects on the immunological changes in astronauts, including heightened pro-inflammatory responses and cell-mediated alterations, or physico-chemical effects inherent of RPM research (complete filling of the culture flasks with medium).

### RPM culture of blood-derived monocytes

The main challenge in studies with monocytes is that they undergo rapid phenotypic changes, including differentiation, during ex vivo culture^56^. Human blood-derived monocytes are mechanosensitive cells, which means that the dynamic cell culture environment has an impact on in vitro cell function and development. The fact that shear stress appears to be important for the physiological function of monocytes is supported by methodological data of Tsubota et al.^57^. They observed that after 16 h in conventional culture, the diapedesis of blood-derived monocytes was irreversibly reduced by 90%. However, the culture under conditions mimicking physiological flow (0.5–0.75 Pa) were sufficient to significantly reduce the impairment of diapedesis in vitro. This observation raises the question of whether the mechanical influence is a friend or foe of the cultured monocytes. Mechanical cues are of vital importance to cells exposed to blood flow or rapidly migrating through tissues^58^. Pressure-sensitive membrane channels, such as Piezo-1, have been linked to mechanosensory functions in monocytes and monocyte-derived macrophages, showing enhanced function to increases in external pressure. Baratchi et al.^59^ demonstrated enhanced monocyte adhesion in response to high shear stresses, with a shear stress-dependent increase in the expression of Piezo-1 channels. Wirthgen et al.^60^ demonstrated that mimicking blood flow in cell culture (shear flow) is associated with the expression of a specific “dynamic” monocyte phenotype. The authors described it as a lower proinflammatory cytokine response to GM-CSF (granulocyte-macrophage colony-stimulating factor), increased anti-inflammatory IL-10 secretion and lower adherence after 24 h. Similarly, 24 h exposure to the RCCS showed reduced levels of TNF and IL-6 in response to LPS stimulation compared to conventional controls^61^. In contrast, Fahy et al.^17^ observed an increase in pro-inflammatory gene expression (*IL6*, *CXCL8*) and protein secretion (TNF, MIP-1α, IL-13) after blood-derived monocytes were exposed to recurrent shear and pressure stress (1 h/day). However, the results are always dependent on the cultivation method and the analysis panel used. Differences exist in type of shear stress (turbulent flow on the RPM versus laminar flow), duration and intensity of the shear stresses and the physico-chemical environment the cells are exposed to, such as completely medium filled flasks in RPM research which are not the standard culture conditions. The current findings illustrated that CD14^+^ PBMCs showed no changes in present surface marker panel when cultured on the RPM compared to static cultured cells. We argue here that the monocytes’ ability to adapt their phenotype to their local mechano-environment could have been impeded by their usual quiescence in the G_0_ phase. It has been found that these cells are less responsive to mechanical cues^62^.

### THP-1 vs. blood-derived monocytes: cell cycle dependence of RPM effects

**Fig. 6:**
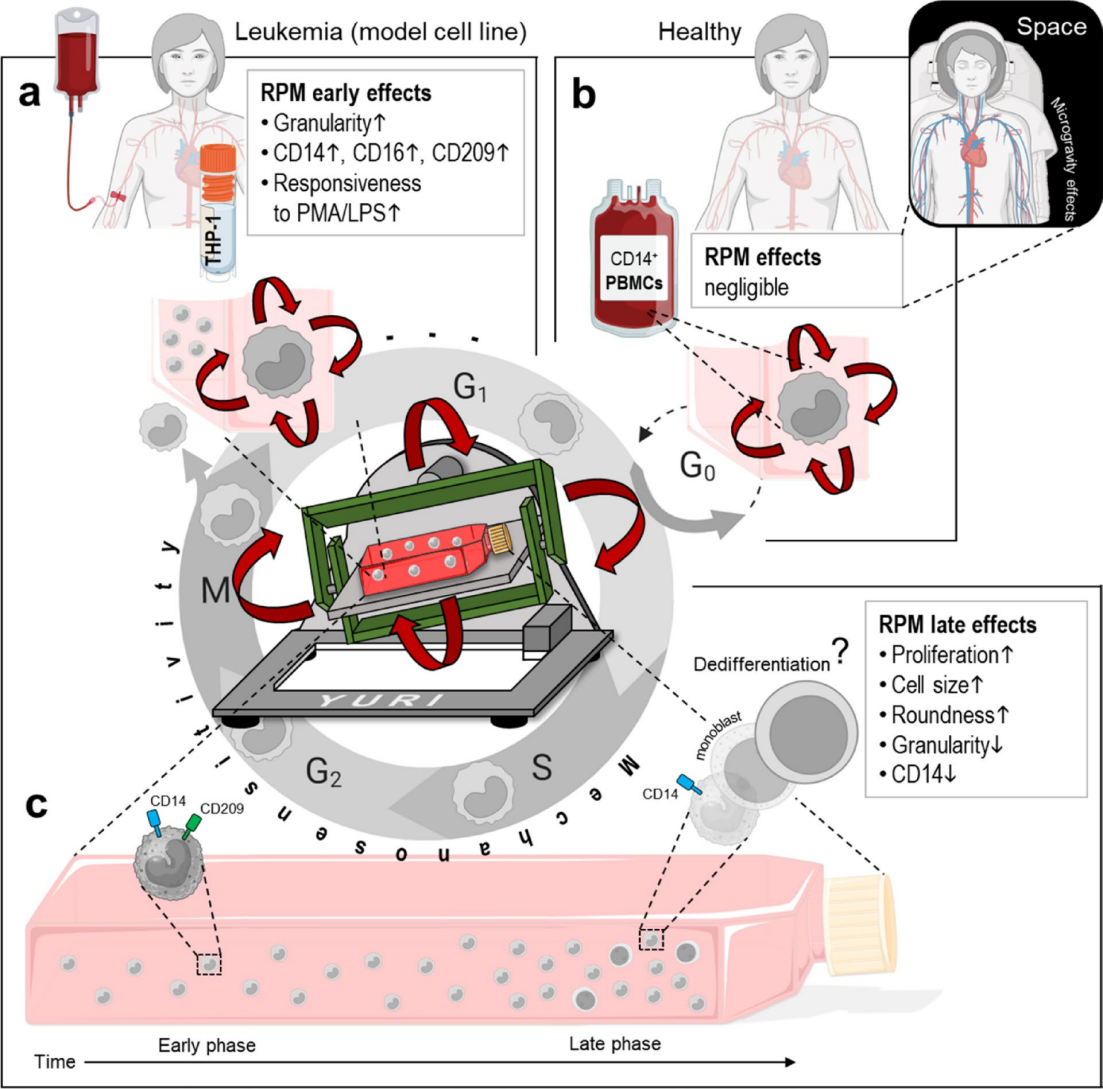
Comparison of RPM effects in subpopulations of (**a**) proliferating leukemic THP-1 monocytes after 3 days and (**b**) non-proliferating monocytes derived from the blood. Only the proliferating cells respond to random positioning, which indicates mechanosensitivity in the progressing cell cycle. Reduced mechanosensitivity in circulating blood-derived monocytes not only indicates resistance to physiomechanical influences (blood pressure, pulse, body movements), but also suggests that microgravity should have no influence on this cell type in astronauts (small image). (**c**) Early and late effects of RPM cell culture on THP-1 cells. The late effects of RPM cell culture show similarities to dedifferentiation of THP-1 monocytes. LPS, lipopolysaccharide; PMA, phorbol 12-myristate 13-acetate. Parts of the figure were drawn using pictures from Biorender.com and Servier Medical Art.

Blood-derived monocytes are often described as having limited potential for proliferation, which complicates in vitro studies. Human-derived monocyte cell lines (i.e., THP-1 cells) are thus often isolated from leukemia patients^63^. Circulating monocytes are generated from proliferating bone marrow hematopoietic stem cells. In healthy humans, the monocytes do not divide during their journey in the bloodstream (G_0_ phase) until they migrate into tissues, where they differentiate locally into functionally distinct macrophages. During disease, such as infections, the numbers and proportions of leukocytes change in part due to the modulation of hematopoiesis and leukocyte mobilization, while in hematological malignancy, the prevalence of immature circulating cells is also increased^64^. Re-entry into the cell cycle could increase the cells susceptibility to mechanical cues, whereas cells that remain quiescent stay insensitive. In this study, THP-1 cells reacted to the RPM with an altered morphology, increased CD14, CD16 and CD209 expression and increased responsiveness to various stimuli (RPM-early phase, Fig. 6a). The blood-derived monocytes showed no response to any of the above-mentioned observations, indicating a certain robustness to the mechanical environment (Fig. 6b).

According to these initial results, the RPM effects appear to be stronger in proliferating cells (THP-1), which indicates mechanosensitivity, than in non-proliferating cells such as blood-derived monocytes. This assumption is also supported by the fact that other proliferating cell types (stem cells, macrophages; cancer cells were generally more strongly influenced by the RPM than healthy cells^65^) react in different ways to random positioning, but not cell cultures with suppressed proliferation. For example, in an experiment with thyroid cancer cells under serum deprivation (a common method for generating a G_1_/G_0_ arrest), the formation of tumor spheroids induced by RPM was not observed. Overgrown proliferation-inhibited cell cultures also respond less to the RPM^9,66^. A reduced mechanosensitivity in non-proliferating, circulating monocytes not only indicates resistance to physiomechanical influences (blood pressure, pulse, body movements), but also suggests that microgravity should have no influence on this cell type in astronauts (Fig. 6b, small figure). In the monocyte differentiation lineage, the THP-1 monocytic cell line corresponds to monoblasts and/or immature monocytes that are characterized by a greater capacity to proliferate and differentiate^67^. According to these initial results, the late RPM effects showed a fast-proliferating culture with a small population that presented a rounder cell shape, increased cell size, decreased granularity and lower expression levels of CD14 (Fig. 6c). Current observations share similarities to dedifferentiation of THP-1 monocytes^68–72^. It is still too early to make a general statement about proliferation-dependent “RPM sensitivity”. However, further studies should provide clarity here in the future. One advantageous property would be the improved mechanical manipulability of highly proliferating cells, e.g. tumor cells, which could reveal new targets not only against leukemia but also against other types of cancer.

### RPM vs. real microgravity: a space research perspective

The latest findings and developments from gravitational and space research with immune cells are regularly summarized in reviews^73–75^. The observation that astronauts exposed to microgravity display impaired immune reactions to pathogens and altered inflammation suggests that these conditions alter the homeostatic properties of immune cells. However, less data is available for monocytes compared to other immune cell types. The monocyte count appears to be relatively stable during short stays in space^76^, while it can increase during long-term missions^77^. Monocyte impairment was noted by a reduced level of pro-inflammatory cytokines (IL-6, TNF and IL-10) in response to LPS stimulation^76^. Although we saw a similar behavior regarding monocyte number in our experiment with THP-1 cells (RPM early phase: stable cell number; RPM late phase: increased cell number), parallels between the in vivo and in vitro data should be drawn with caution. Since blood-derived monocytes do not proliferate, the increase in monocytes in vivo must be related to myeloid progenitor cells. The extent to which this process can be mapped by using a leukemic monocyte cell line is questionable.

Another of our goals was to test the usability of the RPM as a ground-based facility for the preparation of immunological space experiments. In summary, both immortalized and blood-derived monocytes could be cultured on the RPM without losses compared to a static cell culture. However, THP-1 cells responded transiently to RPM-induced mechanical stress, which is absent in real microgravity ex vivo. On the other hand, monocytes in vivo are also exposed to the physiological forces of the circulatory system, which are probably only slightly altered by a stay in weightlessness. The fact that a ground-based simulation facility cannot completely replace a real microgravity experiment is recognized by gravitational biologists^78^. A simulator is always as good as the effects it can imitate. The RPM is rightly used for many space-based biological questions. In the case of mechanosensitive cells, however, the mechanical effects of rotation must not be disregarded. These forces do not occur in microgravity. Whether they have a significant influence on the generated results, whether they lead to similar or completely different results than exposure to weightlessness, must always be evaluated for the respective experiment. In the case of blood cells, random positioning can have a positive effect on the cell culture, as these cells do not remain static in the body but are carried with the surrounding fluid, similar as to what they are on the RPM. However, due to the changing culture conditions over time, it is difficult to attribute observed effects exclusively to “simulated microgravity” in long-term RPM experiments.

## Methods

### Cell lines and cell culture

The human leukemia monocytic THP-1 cell line (passage 7–19) was purchased from Sigma-Aldrich (St. Louis, MO, USA) and kindly gifted from Prof. Heike Walles, University of Magdeburg. THP-1 cells were cultured in RPMI 1640 medium (Life Technologies, Carlsbad, CA, USA) supplemented with 10% fetal calf serum (FCS; Sigma-Aldrich, LOT: 0001661377) and 1% penicillin/streptomycin (Life Technologies) at 37 °C, 5% CO_2_ and a relative humidity of 95%. Cells were routinely checked and imaged using an Olympus CKX53 inverted microscope (Olympus, Tokyo, Japan) in phase contrast mode. Routine cell count was performed using a viable cell counting chamber (Neubauer, Marienfeld, Germany). Cells were maintained between 0.2 and 0.8 × 10^6^ cells/mL before passaging at a maximum confluence of 0.8 × 10^6^ cells/mL, not exceeding 1 × 10^6^ cells/mL. For completely medium filled flask conditions (static/RPM), a cell density of 0.25 or 0.5 × 10^6^ cells were seeded into a T12.5 Blue Vented Screw Cap cell culture flask (Corning Life Sciences, NY, USA) and completely filled with medium. For cell differentiation experiments, a cell density of 0.2 × 10^6^ cells was seeded into a 12-well plate (Greiner Bio-One, Kremsmünster, Austria). For immunofluorescence staining, glass coverslips (Carl Roth, Karlsruhe, Germany) were fixed with sterilized Vaseline (Edeka, Hamburg, Germany) in each well before seeding.

### CD14^+^ PBMCs

Peripheral blood mononuclear cells (PBMCs) were isolated from leukocyte reduction filters (Sepacell RZ-2000; Asahi Kasaei Medical, Tokio, Japan) of 10 healthy adult donors (1 female, 9 males, age range 36–66 years, mean age: 50.5 years). Mononuclear cells were obtained from peripheral blood of healthy donors by centrifugation or through a Ficoll-Hypaque gradient (PAN-Biotech, Aidenbach, Germany). CD14^+^ PBMCs were isolated from PBMCs using CD14-MicroBeads (Miltenyi Biotec, Bergisch Gladbach, Germany) and autoMACS-Pro isolation (Miltenyi Biotec). Monocytes were resuspended in RPMI 1640 (PAN-Biotech) containing 10% fetal bovine serum (Gibco/Life Technologies), 10 µg/mL streptomycin and 10 U/mL penicillin (Life Technologies) and adjusted to 1 × 10^6^ cells/mL monocytes. For completely medium filled flask conditions (static/RPM), a cell density of 1 × 10^6^ cells was seeded into a T12.5 Blue Vented Screw Cap cell culture flask (Corning Life Sciences).

The study was conducted in accordance with the Declaration of Helsinki and was reviewed and approved by the Ethics Committee for Clinical Research of the University of Magdeburg (certificates 26/12, 159/18 and 164/18) and the Ethics Committee of the Medical Association of the State of Saxony-Anhalt (certificate 21/19).

### Random positioning machine

A desktop RPM 2.0 (Yuri, Meckenbeuren, Germany) was used in a HERAcell CO_2_ incubator (Thermo Scientific, Waltham, MA, USA) at 37 °C, 5% CO_2_ without humidification. The RPM was operated in real random mode (two frames rotation) at an average speed of 60°/s (range: 50–70°/s). Before starting the rotation, the cell culture flasks were filled completely with culture medium avoiding bubbles (any remaining bubbles were carefully removed using a pipette tip before closing the culture flask) and closed with Parafilm (Bemis, Fisher Scientific). Static controls were placed in a horizontal formation, next to the RPM in the same incubator at the same environmental conditions.

### Cell differentiation

THP-1 monocytes were differentiated into naïve M_0_ macrophages with 50 ng/mL phorbol 12-myristate 13-acetate (PMA; Sigma-Aldrich) added to the culture medium for 3 days, followed by a 2-day rest period in fresh culture medium. Cells were incubated at 37 °C, 5% CO_2_ and a relative humidity of 95%.

### Flow cytometry

Adherent cells were washed with PBS (Gibco/Life Technologies) and detached from the well plate using Accutase (Sigma-Aldrich) for 10 min at 37 °C, 5% CO_2_ and a relative humidity of 95%. Cell suspensions were collected in centrifugation tubes (Sarstedt) and pelleted by centrifugation at 750×*g* for 5 min at 21 °C and then incubated for 10 min at 4 °C in FACS buffer (1 x PBS + 1% BSA) containing 1:20 dilution FcR blocking reagent (Miltenyi Biotec), followed by incubation for 30 min at 4 °C in FACS buffer containing 1:20 fluorochrome-labeled monoclonal antibodies (eBioscience): CD14 APC (#17-0149-42/61D3), CD16 PerCP eFluor 710 (#46-0168-42/eBioCB16), CD71 FITC (#11-0719-42/OKT9), CD80 PE (#12-0809-42/2D10.4), CD206 eFluor 450 (#48-2069-42/19.2) and CD209 PE cyanine 7 (#25-2099-42/eB-h209). Cells were washed in PBS centrifuged and resuspended in 200 µL PBS before flow cytometry analysis. Flow cytometry acquisition was carried out on a BD FACSCanto II (BD Biosciences), and analysis performed using FlowJo v10.8.1 (https://www.flowjo.com). Unstained cells and compensation beads (UltraComp eBeads, Invitrogen, 01-2222-42) were used to set voltages and single-stain negative and positive controls. The cut-off gating for negative population in the determination of PBMC subsets was determined using fluorescence minus one (FMO) controls. Compensation was set to account for spectral overlap between the six fluorescent channels. Samples were examined by forward scatter area (FSC-A) vs. side scatter area (SSC-A) to select cells (Supplementary Fig. S3). PBMCs divided into subset by marker CD14 vs. CD16. Next, each marker was analyzed separately in each THP-1 sample/PBMC subset.

### Annexin V/PI staining

Cell suspensions were collected and pelleted by centrifugation at 750×*g* for 5 min at 21 °C and then resuspended in 400 µL annexin V staining buffer (BD). A cell density of 1 × 10^5^ cells was transferred to new tubes and stained with 5 µL of fluorescein isothiocyanate (FITC)-conjugated annexin V and 5 µL of propidium iodide. Cells were incubated in the dark for 15 min at room temperature. Finally, 400 µL of staining buffer was added and cells were immediately analyzed with a BD FACSCanto II. The data analysis was performed using FlowJo v10.8.1 (https://www.flowjo.com). For each biological replicate (*n* = 5; unless otherwise specified in the figure legend), 3–5 images were acquired per condition.

### Immunofluorescence microscopy and analysis

For fixation, the suspension cells were collected in centrifugation tubes, centrifuged at 750×*g* for 5 min at 4 °C and fixed with 4% paraformaldehyde (PFA; Carl Roth). Cells were stored in 0.1 M phosphate buffer (PB; Na_2_HPO_4_/NaH_2_PO_4_; Carl Roth, Karlsruhe, Germany) at 4 °C for a maximum of one day before staining. To remove the 4% PFA, the cells were washed three times with 0.1 PB. The cell membranes were permeabilized with 0.2% Tween-20 (Carl Roth, Karlsruhe, Germany) in 0.1 M PB for 15 min. Cells were washed with 0.1 M PB. Washings steps required cell centrifugation (at 300×*g* for 1 min) and careful aspiration of the supernatant. To block non-specific binding sites, 3% bovine serum albumin (BSA, Carl Roth) in 0.1 M PB was used for 1 h at room temperature. Cells were then labeled with 1:400 Phalloidin Alexa Fluor 568 (Invitrogen, #A12380, MA, USA) diluted in 0.1 M PB with 1% BSA for 30 min at room temperature. Cells were washed again and then stained for nuclear DNA. The samples were incubated for 5–10 min with 100 ng/mL DAPI (4′,6-diamidino-2-phenylindole; Thermo Fisher Scientific) in 0.1 M PB, finally washed again with 0.1 M PB and mounted with Fluoromount™ (Sigma-Aldrich).

After staining, the slides were examined using a ZEISS LSM 800 confocal laser scanning microscope (Carl Zeiss, Jena, Germany). To ensure comparability for intensity quantification, all images were acquired with the same settings using the ZEISS Airyscan detector and ZEN v3.10 (Carl Zeiss; https://www.zeiss.com/microscopy/en/products/software/zeiss-zen.html). Airyscan processing settings were optimized for each antibody-wavelength combination and manually applied to the corresponding samples. To ensure nonspecific binding of the secondary antibody and therefore a false negative signal, the secondary antibodies were applied to separate samples of the same condition without the primary antibody. Stacked confocal images for 3D reconstruction of the THP-1 cells were created with ZEN software using the same settings for the RPM and static files.

### Micromer particles

THP-1-derived M_0_ macrophages (0.2 × 10^6^ cells) were incubated with 15 µg/mL micromer-redF (W100 nm, MicroMod) in cell culture medium for 2 h at 37 °C, 5% CO_2_ and a relative humidity of 95%. Cells were washed in PBS, centrifuged and resuspended in 200 µL PBS before flow cytometry analysis.

### Statistical analysis

Statistical analysis was conducted utilizing SPSS Statistics v29 (Statistical Package for the Social Sciences, IBM, Armonk, NY, USA; https://www.ibm.com/products/spss-statistics). To compare samples (biological replicates) from distinct culture conditions, the non-parametric Mann–Whitney U test was used. In instances where there were limited samples (< 5 biological replicates), an independent sample t-test was used. Data is shown as mean ± standard deviation (SD), with individual data points often depicted in most plots. The figure legends provide the sample size for each experiment.

## Conclusion

From a space researchers’ point of view, the different behavior of primary and cell line monocytes on the RPM undoubtedly leads to new hypotheses regarding the behavior and susceptibility of cells under the conditions of “simulated microgravity”. Cell proliferation will certainly receive more attention in future gravitational biology studies, as the connection with the observed effects under altered gravity has not yet been fully clarified. At the same time, observations also teach us not to generalize the results of model experiments too quickly. While differing early and late effects of RPM cell culture were observed in proliferating leukemic THP-1 monocytes, a popular model cell line, the effect of random positioning on non-proliferating healthy blood-derived monocytes was negligible. When using blood-derived monocytes in microgravity experiments, different effects can therefore be assumed than in studies with THP-1 cells, which are often the method of choice in space research due to their robustness. On the one hand, this simplifies analyses, as monocytes in immunocompetent co-culture models on the RPM are not influenced by random positioning and focus of research can be laid on RPM effects on other cell types. On the other hand, the question arises to what extent other non-proliferating cell types, such as finally differentiated cells, confluent or overgrown ones, react sensitive to gravidynamic effects at all. In line, denser cancer cell cultures with lower proliferation showed fewer RPM effects^9^. Furthermore, this could explain the results of CellBox-1, in which a postponement of the rocket launch probably led to an overgrown cell culture and thus prevented the spheroid formation of thyroid tumor cells on the ISS^66,79^. And finally, it would also be good news for astronauts if most fully differentiated, non-dividing body cells were less susceptible to gravitational effects.

Whether the RPM has a positive (dynamic) influence on the cell culture depends strongly on the mechanosensitivity of the cells used in the experiments. This study showed that only leukemic THP-1 monocytes exhibit a transient mechanosensitivity in vitro, where the RPM could affect their function and differentiability. The extent to which this property can be used to develop therapeutic approaches for “abnormal” monocytes must be answered by further research. The RPM can therefore also be a promising tool for biomedical research on Earth.

## Supplementary Information

Below is the link to the electronic supplementary material.

Supplementary Material 1

## Data Availability

The data obtained in this study are shown in the manuscript and in the electronic supplementary materials. The complete raw data are available from the corresponding author (Marcus Krüger) on request.
