# Supplementary material for: Temporary effects of random positioning on the function and plasticity of proliferating monocytes
Source: Sci Rep. 2025 Nov 10;15:39360. doi: 10.1038/s41598-025-26941-x (PMC12603333; doi:10.1038/s41598-025-26941-x)
Supplement: Supplementary file 1 — Supplementary Material 1 [file 41598_2025_26941_MOESM1_ESM.pdf]

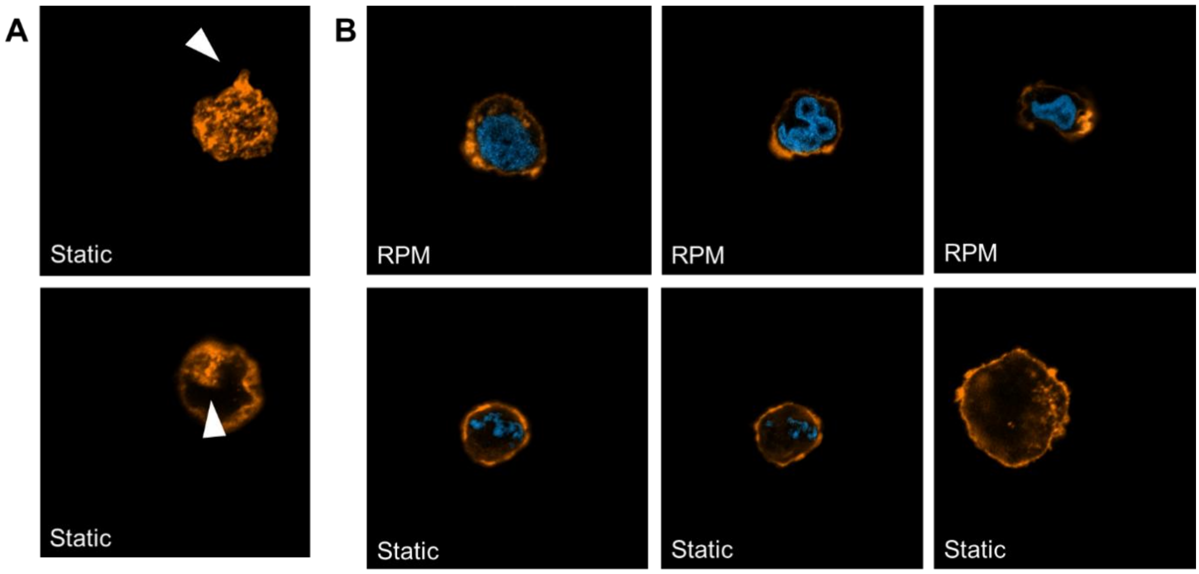

Figure S1. F-actin staining of THP-1 cells under static and RPM conditions (3-day culture). (**a**) Actin accumulation in outward protrusion. A rather exceptional protrusion observed in static cell cultured THP-1 cells. (**b**) IF staining of actin (n = 3). Cells on the RPM show a less diffuse distribution of actin with more localized areas of higher and lower intensity.

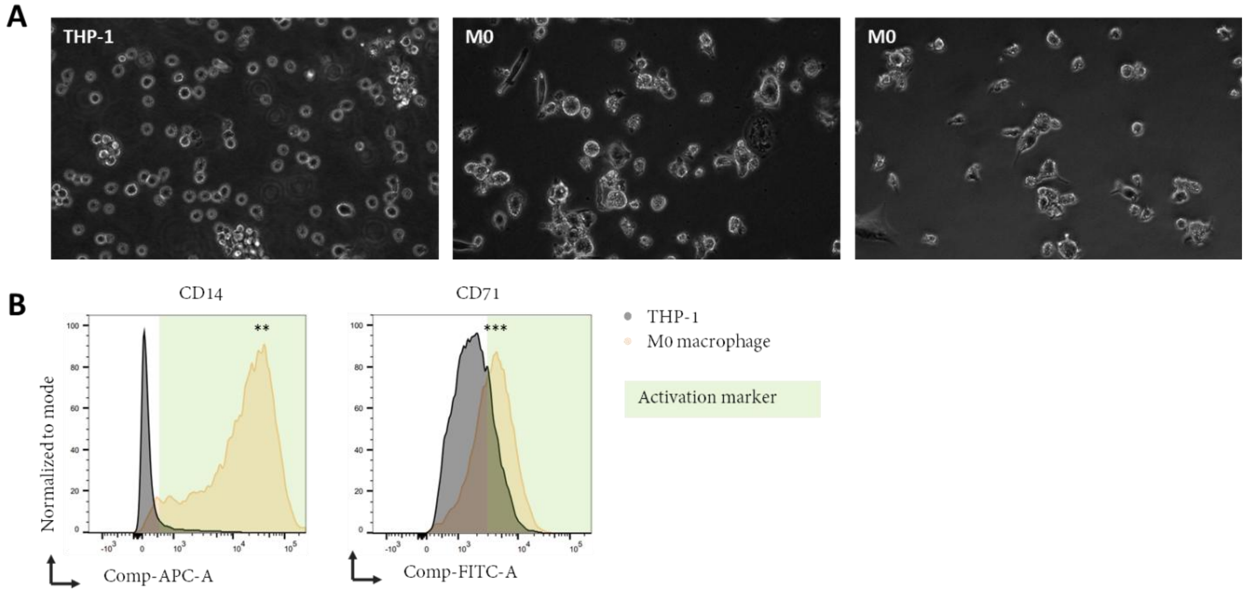

Figure S2. Effects of differentiation on cluster-of-differentiation (CD) markers expressed by THP-1 monocytes and THP-1 derived macrophages. (**a**) The chemical differentiation of THP-1 cells into M_0 macrophages using PMA leads to an optical change in cell morphology under the microscope. (**b**) Expression of the surface markers CD14 and CD71 in monocyte-like THP-1 cells and the resulting macrophage populations (n = 3). Scale bars: 50µm (20×). Independent sample t-test ** $p \leq 0.01$, *** $p \leq 0.001$.

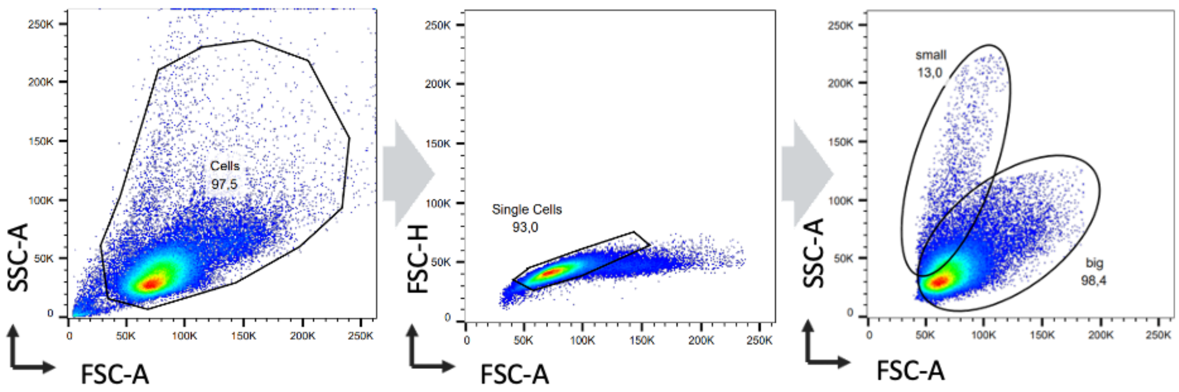

Figure S3. Gating strategy for isolation of a pure population of individual cells. A combination of scatter parameters (FSC and SSC) and pulse geometry (FSC-H vs. FSC-A) were used to exclude cell debris, clumps (doublets) and dead cells. *FSC-H: forwards scatter – Height; FSC-A: forward scatter – Area; SSC-A: sideward scatter – Area.*
